# Therapy with Immune Checkpoint Inhibitors for Solid Tumors in Patients with Preexisting Systemic Autoimmune Diseases

**DOI:** 10.3390/jcm14217765

**Published:** 2025-11-01

**Authors:** Sara Elena Campos Ramírez, Pablo Gómez Mugarza, Paula Gomila Pons, Carmen Blanco Abad, María Pilar Felices Lobera, Sofía Elena Ruffini Egea, Pilar Rivero Sobreviela, Luis Gallart Caballero, Paula Morillas Martínez, Ana María Comín Orce

**Affiliations:** Medical Oncology, University Hospital Miguel Servet, 50009 Zaragoza, Spain

**Keywords:** immune checkpoint inhibitors, solid tumors, systemic immune diseases, efficacy, safety, flare, lung cancer, melanoma, cutaneous carcinoma, renal cancer

## Abstract

**Background:** Patients with systemic autoimmune diseases (SAID) are at a higher risk of developing neoplasms, such as solid tumors and hematologic malignancies. Chronic stimulation of the immune system and some treatments for these diseases increase the risk of developing solid tumors. Also, it is known that patients with SAID are usually excluded from clinical trials, but immune checkpoint inhibitors (ICI) are still used in these patients in everyday practice. **Objectives:** The objective of this article is to review the most up-to-date and robust literature on the use of ICI in patients with SAID for the treatment of solid tumors to obtain information on the efficacy and safety of these drugs in this subgroup of patients. **Methods:** A literature review was performed through international databases that included PubMed, Medline, Scopus, and Google Scholar. Articles about the use of ICI for solid tumors in patients with SAID were included; the types of articles included were retrospective studies, systematic reviews, and meta-analyses. A summarized descriptive analysis was performed about the efficacy and safety of ICI treatment for the main solid tumors (lung, melanoma, and other cutaneous malignancies, as well as renal and urothelial carcinoma). **Conclusions**: In general, it seems that ICI treatment is safe in patients with asymptomatic SAID. Close follow-up with a multidisciplinary team should be performed when ICI therapy is prescribed. A substitution of selective immunosuppressants (SIM) in place of nonselective immunosuppressants (NSIM) in asymptomatic patients is recommended before the initiation of ICI.

## 1. Introduction

Systemic autoinflammatory diseases (SAID) include a heterogeneous group of disorders where there is a dysfunction of the immune system of the patient, leading to an overresponse of the innate and adaptive mechanisms of immunological response [1]. An elevated risk of both hematological and non-hematological malignancies has been observed across various autoimmune diseases. Immune checkpoint inhibitors (ICI), such as anti-cytotoxic T-lymphocyte antigen 4 (anti-CTLA-4) and anti-programmed cell death protein 1 or ligand (anti-PD-1/PD-L1), have become one of the key components of the treatment of solid tumors in advanced and early disease settings. The use of ICI is known to be a risk factor for the development of autoimmune disorders and exacerbation of preexisting SAID; moreover, patients with SAID have been generally excluded from clinical trials [2,3].

In 2020, an ESMO (European Society of Medical Oncology) recommendation guideline was published for the approach for these patients, establishing a two-step strategy that includes the replacement of nonselective immunosuppressants (NSIM), like corticosteroids, methotrexate (MTX), azathioprine (AZT), mycophenolate mofetil (MMF), and others,, with selective immunosuppressants (SIM), such as infliximab (IFX), tocilizumab (TCZ), vedolizumab (VDZ), and others, in patients with asymptomatic or oligosymptomatic SAID. After a period of induction, if there is no exacerbation of the symptoms, SIM can be administered in combination with ICI therapy [3].

Another widely recommended practice is close monitoring of flare development by a multidisciplinary team for the prevention or early detection of events that could potentially lead to high comorbidity or mortality [3,4].

In this article, we provide a comprehensive review of the available literature, with particular attention to the most frequently reported malignancies. We further examine the evidence regarding the efficacy and safety of ICI and analyze the range of strategies implemented to mitigate morbidity and mortality associated with their use in patients with SAID.

## 2. Materials and Methods

An extensive literature review was conducted to identify studies evaluating the use of immune checkpoint inhibitors (ICI) in patients with pre-existing systemic autoimmune diseases (SAID). We performed research through international databases (PubMed/MEDLINE, Scopus, and Google Scholar). We included retrospective studies, systematic reviews, and meta-analyses published from January 2010 to the date of August the 1st of 2025.

The search was performed using keywords related (but not limited) to “immune checkpoint inhibitors,” “autoimmune diseases,” “solid tumors,” “lung cancer,” “melanoma,” “cutaneous malignancies,” “renal cancer,” “efficacy,” and “safety.”

Eligibility criteria included original studies, such as retrospective studies (cohort studies), systematic reviews, and meta-analyses that reported data on the safety, efficacy, or management strategies related to ICI for the treatment of solid tumors (mainly, but not limited to, lung cancer, melanoma, squamous carcinoma of the head and neck, and kidney carcinoma) in patients with preexisting SAID. This study excluded case reports, expert opinions, and original articles that were part of any of the meta-analyses included.

Data was extracted with the aim of collecting information on patient demographics, type of autoimmune disease, cancer type, ICI regimen, clinical outcomes, immune-related adverse events, and management strategies. This data was later synthesized through descriptive and qualitative representations.

## 3. Results

### 3.1. Lung Cancer

In 2024, a Japanese study analyzed 229 patients with advanced or recurrent non–small cell lung cancer (NSCLC) and preexisting systemic autoimmune diseases (SAID) treated with immune checkpoint inhibitors (ICI; anti–PD-1/PD-L1 and anti–CTLA-4) as a monotherapy or in combination with chemotherapy (ChT) between 2010 and 2020. The most frequent SAID were rheumatoid arthritis (RA), thyroiditis, and Sjögren’s syndrome (SS). The SAID flare rate was 25.4% (18 patients) of whom 10 patients required additional treatment with nonsteroidal immunomodulators (NSIM). Only one patient discontinued ICI due to a SAID flare. The objective response rate (ORR) and disease control rate (DCR) were 33.8% (95% confidence interval [CI], 23.2–46.1%) and 59.1% (95% CI, 46.8–70.5%), respectively. Median overall survival (mOS) was 16.5 months (95% CI, 14.3–NE) [5].

In 2023, a retrospective cohort study included 10,963 patients with either NSCLC or small cell lung cancer (SCLC), at both local and metastatic stages, who were treated at Johns Hopkins Hospital. Of these, 454 patients had a preexisting autoimmune rheumatic disease (ARD), such as RA (114 patients), psoriasis (61), SS (54), polymyalgia rheumatica (PR, 33), systemic lupus erythematosus (SLE, 24), and others. The median overall survival (mOS) for the entire population with SAID was not reached by this cohort, and among metastatic patients with SAID, mOS was 1.2 years; this effect was observed regardless of the specific ARD. Patients who received NSIM, such as glucocorticoids, prior to ICI initiation had worse outcomes compared with those treated with monoclonal antibodies for SAID (HR 0.6, *p* = 0.012) [6].

In a study by Leonardi et al. [7], among 56 NSCLC patients with coexisting SAID, 23% experienced worsening of autoimmune symptoms (flares), mostly mild to moderate in severity. Patients who initiated cancer treatment while their autoimmune disease was active exhibited a higher frequency of flares (50% versus 18%), whereas concomitant immunosuppressive therapy did not influence the flare rate (36% with active immunosuppressive therapy versus 20% without). The incidence of immune-related adverse events (irAEs) was 38%, with 71% being grade 1 or 2. The ORR was 22%, and the DCR was 53%, with no correlation found between the use of NSIM prior to ICI initiation and clinical outcomes.

A meta-analysis of nine studies involving 250 NSCLC patients with various preexisting SAID treated with ICI reported a flare incidence of 23% in the lung cancer subgroup. The presence of SAID was associated with a higher risk of both exacerbation of autoimmune symptoms and de novo irAEs. In this analysis, SAID were also linked to improved tumor response (risk ratio [RR] 1.56; 95% CI, 1.19–2.04) [8].

A summary of the main findings from these studies is shown in Table 1 [5,6,7,8].

Overall, the presence of SAID appears to be associated with a higher risk of immune-related adverse events (irAEs) and disease flares, as supported by studies reporting that 30–40% of patients develop irAEs and 20–30% experience disease flares [9,10]. In two previous studies, patients with lung cancer and preexisting SAID exhibited improved clinical outcomes with ICI therapy, including significantly longer overall survival (OS) and progression-free survival (PFS) compared with patients without such conditions [6,11]. Nevertheless, these findings are not entirely consistent, as other studies reported no significant differences in survival or treatment response between patients with and without preexisting autoimmune diseases, highlighting the variability and complexity of immune-related interactions in this population [12,13].

### 3.2. Melanoma

In a Dutch cohort study of 4367 patients with advanced melanoma treated with ICI, patients were stratified according to the presence of SAID (*n* = 415). The incidence of grade ≥ 3 irAEs ranged from 30% to 48%, with the highest rates observed in patients receiving combination therapy. No statistically significant differences were found compared with patients without SAID. In most cases, irAEs manifested in the same organ system affected by the underlying autoimmune condition. The OS did not differ significantly between patients with and without SAID. Notably, consistent with findings in lung cancer, patients with a history of prior immunosuppressive therapy had worse median OS (12–13 months) compared with those without such treatment (13–23 months), although this association was no longer significant after adjustment for confounding variables [14].

In a study by Plaçais et al. [15], 110 patients with stage III–IV melanoma and preexisting SAID treated with ICI were matched to 354 controls. The incidence of any-grade irAEs was 72% in patients with SAID, with 57% experiencing grade ≥ 3 events. Most irAEs reported were autoimmune thyroiditis, psoriasis, rheumatoid arthritis, and vitiligo. Patients with SAID had a higher risk of all-grade, grade ≥ 3, and multiple irAEs, particularly among those receiving anti–PD-1 monotherapy or combined anti–PD-1/anti–CTLA-4 therapy. No differences were observed in OS, irAE-related mortality, or when stratified by prior immunosuppressive therapy. The estimated OS at 24 months was 64.8% in cases and 45.9% in controls, with no statistically significant difference between the two groups.

In another study of 30 patients with advanced melanoma (stages IIIC and IV) with preexisting SAID treated with ipilimumab (anti–CTLA-4) the flare rate was 27%, of which 33% were grade ≥ 3. One treatment-related death occurred: a patient with prior psoriasis on active NSIM therapy developed colitis that led to death within two days of symptom onset. Grade ≥ 3 irAEs were observed in 33% of patients. Compared with previous studies, the time from ICI initiation to irAE onset was shorter (1.5 months versus 3–6 months) [15,16].

In a study of 119 patients with melanoma treated with anti–PD-1 therapy (pembrolizumab or nivolumab), 52 patients had preexisting SAID. The flare rate was 38%, typically as an exacerbation of preexisting symptoms rather than an extension of disease manifestation. Among patients presenting a flare, 60% had symptomatic SAID before anti–PD-1 therapy, and 50% of these were receiving NSIM. No treatment-related deaths were observed. The objective response rate (ORR) was 33%, median progression-free survival (mPFS) was 6.2 months, and the median OS was not reached. No difference in ORR was observed between patients who developed flares and those who did not (35% versus 31%). As in other studies, patients on NSIM therapy before anti–PD-1 initiation had a worse ORR than those not on NSIM (15% versus 44%), and this difference remained significant after adjustment for confounding factors [17].

In 2020, Brown, LJ et al. conducted a study of 55 patients with advanced melanoma (stages III–IV) and preexisting SAID treated with combination anti–PD-1 (nivolumab) plus anti–CTLA-4 (ipilimumab). The flare rate was 33%; 89% occurred during initial combination therapy and 11% during maintenance with nivolumab. Consistent with the ipilimumab monotherapy study [16], symptom onset occurred early, at a median of 19 days from ICI initiation. Two patients required intensive care due to severe flares of ulcerative colitis. Among patients with flares, 50% had previously symptomatic SAID (versus 29% without) and 39% were on active immunosuppressive therapy (versus 26% without). The ORR for the overall population was 55%, with differences observed between patients receiving NSIM versus no NSIM (46% versus 57%). This difference was also reflected in 12-month PFS (15% versus 48%) [18].

A summary of the main findings from these studies is presented in Table 2 [14,15,16,17,18].

### 3.3. Other Less Studied Carcinomas

Avelumab has been approved for the treatment of advanced Merkel cell carcinoma (MCC) in first-line and second-line settings; real-world evidence demonstrates an ORR of 29.1% to 58% and a mPFS of 8.1 to 24.4 months [19]. Other anti-PD1s have been studied, such as pembrolizumab and nivolumab, with high and durable responses. So far, the published data include a case report of a patient with preexisting myasthenia gravis who was diagnosed with MCC and successfully treated with pembrolizumab. The patient experienced autoimmune hepatitis, which was managed by switching from MMF to cyclosporine A, allowing for the continuation of pembrolizumab treatment with a partial response [20]. No prospective studies have been developed regarding this specific subject. More evidence in this rare cancer comes from multiple carcinoma studies in patients with SAID where MCC patients were included.

Regarding squamous cutaneous carcinoma (SCC), to date, there is only a brief American Society of Clinical Oncology (ASCO) communication reported so far; this evaluated the safety and efficacy of cemiplimab for the treatment of advanced SCC. In the study there were 11 patients included with SAID, the ORR in the group of patients with SAID and transplant organ recipients (*n* = 6) was 47%, and the only adverse event reported in this subgroup was organ transplant rejection [21]. There is another case report published in 2023 of a patient with vasculitis and SCC who was treated with cemiplimab, presenting a partial response to treatment and no toxicity associated with ICI [22].

There are no prospective or retrospective studies with patients with SAID and genitourinary (GU) malignancies exclusively; evidence of the use of ICI in this group of patients comes from studies that included patients with GU cancer. A summary of studies published to date of patients with SAID who were treated with ICI due to multiple types of carcinomas is shown in Table 3 [23,24,25,26,27].

## 4. Discussion

The use of immune checkpoint inhibitors (ICI) in patients with systemic autoimmune diseases (SAID) has been extensively studied, mainly in patients with lung cancer and melanoma. Available data are primarily from retrospective studies and meta-analyses, with no prospective studies published to date. In lung cancer, flare rates range from 20% to 30%, usually presenting as mild to moderate exacerbations. Asao et al. [5] demonstrated that patients with preexisting SAID can benefit from immunotherapy, with survival outcomes comparable to those without autoimmune conditions, although with a slight increase in immune-related adverse events (irAEs). Ghanem et al. [6] reported that patients with autoimmune rheumatic diseases experienced even better clinical outcomes, suggesting a potential favorable immunologic synergy. In contrast, Leonardi et al. [7], in an earlier prospective study, observed a higher incidence of immune-mediated toxicities and autoimmune flares, though most were manageable and did not compromise antitumor efficacy. Finally, Aung et al. [8], through a comprehensive meta-analysis, confirmed that immunotherapy in this subgroup is generally safe and effective, with response rates and survival comparable to patients without autoimmunity. Collectively, these studies support the cautious but justified use of ICIs in NSCLC patients with autoimmune disorders, emphasizing the importance of close monitoring to balance oncologic benefit against immune-related toxicity.

In melanoma, the flare rate is approximately 30%, comparable to that observed in lung cancer [14,15,16,17,18]. Notably, patients receiving combination therapy with anti–CTLA-4 and anti–PD-1 agents experienced a higher incidence of de novo irAEs compared with those treated with anti–PD-1/PD-L1 monotherapy. This aligns with safety data from ICI studies, which consistently show that combination regimens are associated with increased rates of irAEs of any grade and grade ≥ 3 events. In addition to higher frequency, the time to irAE onset is usually shorter with combination therapy compared to monotherapy. This is relevant for patient education and physician monitoring of side effects [14,15,18,28,29,30].

Conflicting results exist regarding the correlation between preexisting SAID and risk of flares or de novo irAEs. Some studies report an association, whereas others do not. Numerically, patients with preexisting SAID appear more likely to experience both autoimmune exacerbations and de novo irAEs. However, these events are typically mild to moderate and rarely lead to treatment discontinuation.

Although ICI use in patients with SAID is generally feasible and safe, several considerations should be addressed. First, the presence of active SAID symptoms should be assessed before initiating ICI therapy, as this may necessitate concomitant immunosuppressive treatment and could increase the severity of flares, morbidity, and mortality [3,17]. Notably, studies report that deaths related to ICI-associated exacerbations often occur in patients with active SAID symptoms, such as ulcerative colitis [15,18]. Therefore, both the presence and severity of active symptoms should be considered when planning treatment.

Regarding prior immunosuppressive therapy, and in accordance with ESMO recommendations [3], patients—whether with active disease or not—should preferably receive selective immunomodulators (SIM) rather than nonselective immunomodulators (NSIM), such as corticosteroids, which are associated with poorer oncologic outcomes. Baseline corticosteroid use at doses ≥ 10 mg/day of prednisone equivalent has been consistently linked to reduced response rates, shorter progression-free survival (PFS), and overall survival (OS) in patients treated with ICIs. The negative impact of corticosteroids depends on timing, indication, and dosage; non-palliative low-dose exposure (<10 mg/day) appears less detrimental, whereas higher or prolonged courses may compromise ICI efficacy [3,31,32].

The ESMO strategy proposed in 2020 recommends withdrawing NSIM and introducing an induction period with SIM prior to initiating ICI therapy [3]. Although this may not always be feasible, joint follow-up by oncologists and the specialists managing SAID should be established from the first patient contact and continue before, during, and after ICI therapy [3]. Multidisciplinary follow-up should be particularly strict during the first three to six months after ICI initiation, as complications are more frequent in this period [15,16,33].

Special caution is warranted regarding ICI type. Evidence shows that anti–CTLA-4 combined with anti–PD-1 or anti–PD-L1 carries a higher-risk safety profile compared with anti–CTLA-4 monotherapy, which in turn is less toxic than anti–PD-1/PD-L1 monotherapy [15,16,17,18,34].

Given the limited evidence for other tumors (e.g., renal or cutaneous squamous cell carcinoma), ICI use in these patients should be individualized and guided by data from more extensively studied tumor types.

We believe that the discrepancies found in some studies may be due to the highly heterogeneous nature of the SAID populations in each study (e.g., use of NSIM, asymptomatic disease, etc.), both within each study and compared to others, which may lead to variations in outcomes. Therefore, we consider it crucial that, from now on, patients with SAID be included in clinical trials using more homogeneous criteria that can be extrapolated to real-world clinical practice.

**Figure 1:**
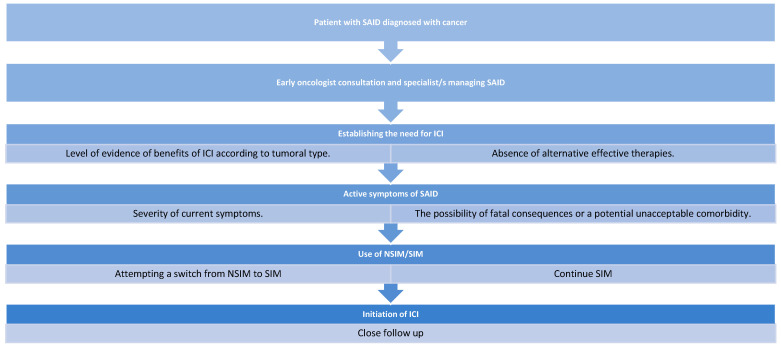
Schematic summary of factors to consider in ICI treatment for patients with SAID. SAID: Systemic autoimmune diseases, ICI: Immune checkpoint inhibitors, SIM: Selective immunosuppressants, NSIM: Non-selective immunosuppressants.

In summary, the management of patients with preexisting SAID requiring ICI therapy requires a careful balance of risks and benefits. Patients should be fully informed about potential consequences and available alternatives before initiating or discontinuing treatment. Figure 1 presents a recommended schematic for managing patients with SAID who require ICI therapy.

The main limitation of this study is that it represents a narrative review rather than a systematic review or meta-analysis, which may introduce selection bias in the inclusion of studies. Additionally, all the analyzed data are derived from retrospective studies, limiting the ability to establish causality and increasing the potential for confounding factors and reporting bias. We consider that, given the evidence from retrospective studies (reflecting real-world clinical practice) showing that, in general, the efficacy of ICI treatment is not affected in patients with SAID, these patients should begin to be included in clinical trials (following certain specific criteria already known to not influence ICI efficacy, such as low-dose corticosteroids and asymptomatic SAID, among others).

## 5. Conclusions

The use of ICI in patients with SAID is generally safe, involving a slightly higher risk of exacerbation and irAE. Among the factors to consider before initiating ICI therapy in this population are the presence of SAID symptoms, the use of non-immunosuppressive or immunosuppressive medications, the type of ICI therapy to be used, and the possibility of close follow-up from both the oncologist and the specialist involved in the management of the SAID.

## Figures and Tables

**Table 1:** Summary of principal findings in studies on patients with lung cancer and pre-existing SAID treated with ICI.

Author	Year	Type of Study	Total Patients	Tumor	SAID	Safety Data	Efficacy Data	Comments
Asao, T., et al.	2024	Retrospective cohort study	229	Advanced or recurrent NSCLC	RA, thyroiditis, SS	Flare rate 25.4%.	DCR 59.1%. mOS 16.5 months. DCR and PFS: no data	
Ghanem, P., et al.	2023	Retrospective cohort study	10,963	Local or advanced NSCLC or SCLC	Multiple	Flare rate 29%.	mOS not reached. mOS 1.2 years in stage IV patients. DCR, ORR, and PFS: no data	Worse results in patients with NSIM treatment before the ICI therapy
Leonardi, G., et al.	2018	Retrospective cohort study	56	Advanced NSCLC	Multiple	Flare rate 23%. 14% discontinued due to toxicity irAR rate: 38%	ORR: 22% DCR 53%. OS and PFS: no data	Patients with active symptoms had more risk of flare
Aung, W.Y., et al.	2023	Meta-analysis	11,567	Advanced NSCLC	Multiple	Flare rate 23%	ORR 24% DCR, OS, and PFS: no data.	Increased disease control in patients with SAID

*RA: rheumatoid arthritis, DCR: disease control rate, mOS: median overall survival, NSIM: nonselective immunosuppressant, ICI: immune checkpoint inhibitors, ORR: overall response rate, SAID: systemic autoimmune diseases.*

**Table 2:** Summary of principal findings in studies on patients with melanoma and cutaneous cancer and pre-existing SAID treated with ICI.

Author	Year	Type of Study	Total Patients	Tumor	SAID	Safety Data	Efficacy Data
Kumar, R., et al.	2022	Retrospective cohort study	40	Melanoma, lung cancer, breast cancer, and others	Multiple	irAEs incidence: Anti-CTLA4: 30% Anti-PD1: 17%. Combination therapy: 48%.	ORR: 10–40% mOS: 13 months DCR and PFS: no data
Plaçais, L., et al.	2022	Case–control matched study	110	Melanoma	Multiple	irAEs risk of patients with SAID vs. no SAID: Anti-PD1: OR 2.4. Anti-CTLA4: OR 1.82. Combination therapy: OR 2.31. Flare rate: 30%.	24-month OS: 64.8%. ORR, DCR, and PFS: no data
Johnson, D.B., et al.	2016	Retrospective cohort study	30	Melanoma (ipilimumab in monotherapy)	Multiple	Flare rate: 27%. Grade ≥ 3 irAE: 33%. 1 death related to irAE (colitis).	mOS: 12.0 months. mPFS: 3.0 months. ORR: 20%. DCR: 30%.
Menzies, A.M., et al.	2019	Retrospective cohort study	119	Melanoma (nivolumab or pembrolizumab in monotherapy)	Multiple	Flare rate: 38%. irAEs rate: 29%, 10% grade ≥ 3.	ORR: 33%. mPFS: 6.2 months. mOS not reached. PFS: no data.
Brown, L.J., et al.	2020	Retrospective cohort study	55	Melanoma (combination therapy)	Multiple	Flare rate: 33%. irAEs rate: 67%, 38% grade ≥ 3.	ORR: 55%. mPFS: 10 months. DCR and PFS: no data

*irAE: immune-related adverse events, ORR: overall response rate, mOS: median overall survival, SAID: systemic autoimmune diseases, OR: odds ratio, mPFS: median progression-free survival, DCR: disease control rate.*

**Table 3:** Studies of patients with SAID treated with ICI for various types of carcinomas.

Study	Year	Study Type	Total Patients	Main Tumors Studied	SAID	Safety Data	Efficacy Data
Cai, Q., et al.	2022	Meta-analysis (14 studies)	8716	NSCLC, melanoma, RCC, urothelial carcinomas.	Multiple	Any-grade irAEs rate: 29–100% vs. 9.5–96.4% (SAID versus no SAID) Grade ≥ 3 irAEs: 7.1–56% versus 4.1–45.4% (SAID versus no SAID) RR: 1.74 (95% CI, 1.27–2.37), higher risk in the SAID group.	No difference in PFS (HR: 1.09, 95% CI, 0.96–1.24). No difference in OS (HR: 1.07, 95% CI, 0.94–1.22).
Liu, X., et al.	2024	Meta-analysis (23 studies).	643	Melanoma, NSCLC, urological cancer.	Multiple	irAEs rate: 64% (95% CI, 55–72%) Combined flare rate: 41% (95% CI, 31–50%)	ORR: 30% (95% CI, 15–46%). DCR: 44% (95% CI, 24–66%).
Xie, W., et al.	2020	Meta-analysis (14 studies)	619	Melanoma, NSCLC, RCC, urothelial cancer.	Multiple	irAEs: 33% (95% CI, 24–42%) Flare rate: 35% (95% CI, 29–41%)	ORR: 30% (95% CI, 22–39%). DCR: 40% and 39% (95% CI, 23–54%). mOS: 10.5–22.5 months mPFS: 3.0–14.4 months.
Yamaguchi, A., et al.	2021	Meta-analysis (38 studies)	206	NSCLC, melanoma, RCC, urothelial carcinoma.	Multiple	irAEs rate: 62.1% (SAID) versus 51.9% (no SAID). OR = 2.14 (95% CI, 1.58–2.89)	ORR, DCR, OS, and PFS: No data
Le, J., et al.	2025	Meta-analysis (52 studies).	No data	NSCLC, melanoma	Multiple	irAEs incidence 0.610 (95% CI, 0.531–0.686). Flare incidence 0.295 (95% CI, 0.248–0.343).	ORR pooled rate: 0.333 (95% CI, 0.262–0.407). DCR pooled rate: 0.554 (95% CI, 0.426–0.678). No difference in OS (SAID versus no SAID) HR: 0.97 (95% CI, 0.88–1.08). No difference in PFS (SAID versus no SAID) HR 0.79 (95% CI, 0.52–1.20).

*NSCLC: non-small cell lung cancer, RCC: renal cell cancer, irAE: immune-related adverse event, RR: relative risk, CI: confidence interval, ORR: overall response rate, DCR: disease control rate, mOS: median overall survival, mPFS: median progression-free survival, SAID: systemic autoimmune diseases.*

## Data Availability

No new data were created or analyzed in this study. Data sharing is not applicable to this article.

## Abbreviations

The following abbreviations are used in this manuscript:

Anti-CTLA4	Anti-cytotoxic T-lymphocyte antigen 4
Anti-PD-1	Anti-programmed cell death protein 1
Anti-PD-L1	Anti-programmed cell death protein 1 ligand
ASCO	American society of clinical oncology
AZT	Azathioprine
ChT	Chemotherapy
CI	Confidence interval
DCR	Disease control rate
ESMO	European Society of Medical Oncology
GU	Genitourinary
IFX	Infliximab
ICI	Immune checkpoint inhibitors
irAE	Immune-related adverse events
MCC	Merk cell carcinoma
MMF	Mycophenolate mofetil
mOS	Median overall survival
MTX	Methotrexate
NSCLC	Non-small cell lung cancer
NSIM	Non-selective immunosuppressants
ORR	Overall response rate
RA	Rheumatoid arthritis
SAID	Systemic autoimmmune diseases
SCC	Squamous cell carcinoma
SIM	Selective immunosuppressants
SS	Sjögren’s syndrome
TCZ	Tocilizumab
VDZ	Vedolizumab
